# Nitric oxide synthase mediates PC12 differentiation induced by the surface topography of nanostructured TiO_2_

**DOI:** 10.1186/1477-3155-11-35

**Published:** 2013-10-11

**Authors:** Margherita Tamplenizza, Cristina Lenardi, Elisa Maffioli, Simona Nonnis, Armando Negri, Stefania Forti, Elisa Sogne, Silvia De Astis, Michela Matteoli, Carsten Schulte, Paolo Milani, Gabriella Tedeschi

**Affiliations:** 1 CIMAINA and Dipartimento di Fisica, Università degli Studi di Milano, via Celoria 16, Milano 20133, Italy; 2 D.I.V.E.T., Università degli Studi di Milano, via Celoria 10, Milano 20133, Italy; 3 Fondazione Filarete, viale Ortles 22/4, Milano 20139, Italy; 4 Dipartimento di Biotecnologie Mediche e Medicina Traslazionale, Università degli Studi di Milano, via Vanvitelli 32, Milano 20133, Italy; 5 Clinical and Research Center Humanitas, via Manzoni 56, Rozzano, Italy

**Keywords:** Nanostructured titanium oxide film, PC12, Tyrosine nitration, NOS

## Abstract

**Background:**

Substrate nanoscale topography influences cell proliferation and differentiation through mechanisms that are at present poorly understood. In particular the molecular mechanism through which cells 'sense’ and adapt to the substrate and activate specific intracellular signals, influencing cells survival and behavior, remains to be clarified.

**Results:**

To characterize these processes at the molecular level we studied the differentiation of PC12 cells on nanostructured TiO_2_ films obtained by supersonic cluster beam deposition.

Our findings indicate that, in PC12 cells grown without Nerve Growth Factor (NGF), the roughness of nanostructured TiO_2_ triggers neuritogenesis by activating the expression of nitric oxide synthase (NOS) and the phospho-extracellular signal-regulated kinase 1/2 (pERK1/2) signaling. Differentiation is associated with an increase in protein nitration as observed in PC12 cells grown on flat surfaces in the presence of NGF. We demonstrate that cell differentiation and protein nitration induced by topography are not specific for PC12 cells but can be regarded as generalized effects produced by the substrate on different neuronal-like cell types, as shown by growing the human neuroblastoma SH-SY5Y cell line on nanostructured TiO_2_.

**Conclusion:**

Our data provide the evidence that the nitric oxide (NO) signal cascade is involved in the differentiation process induced by nanotopography, adding new information on the mechanism and proteins involved in the neuritogenesis triggered by the surface properties.

## Background

Cellular behavior *in vivo* and *in vitro* is heavily influenced by the mechanical, biochemical and topographical properties of the extracellular environment where cells grow [1-3]. In the last two decades a rapidly increasing amount of data suggested that the modulation of topographical and chemical cues at the nanoscale plays a relevant role in determining cell adhesion, proliferation and differentiation [4]. Cells in their natural environment interact with extracellular matrix (ECM) components structured at the nanometer scale [5] and they respond to nanoscale features when grown on synthetic substrates [6-10].

In order to elucidate the role of substrate topography and to fabricate smart biocompatible interfaces capable of mimicking the physiological conditions of the extracellular environment, a large number of studies have been devoted to the investigation of cell interactions with artificially produced nanostructures such as pits, pillars, grooves, dots or random patterns obtained by chemically or physically etching of metallic, semiconducting and polymeric surfaces [9,11-13]. The fabrication strategies employed to create synthetic substrates with tailored topography at the nano- and microscale are essentially based on hard and soft lithography and thus quite inefficient for the reproduction of the random morphology and the hierarchical organization typical of the ECMs [5].

Particular attention has been concentrated on the effect of micro- and nanoscale topography on neuronal growth and differentiation with a focus on axonal guidance and neuronal regeneration [13-15]. It was observed that, in addition to serving as contact guidance, topography often works synergistically with the appropriate biochemical cues to regulate differentiation as well as proliferation [11]. Experimental results suggest that a combination of spatial, chemical and mechanical inputs, together with the genetic properties and protein expression in the cell, control the shape and functions of neuronal cells during neuron growth and differentiation [12,16]. Despite the large amount of data, many fundamental aspects remain to be clarified and, in particular, the molecular mechanism through which cells 'sense’ and adapt to the surface of the adhesion and activate specific intracellular signals influencing cell survival, proliferation and differentiation.

The rat pheochromocytoma (PC12) cell line has been widely used as a neuronal model system to study neuronal differentiation and specific growth factor signaling mechanisms. When stimulated with nerve growth factor (NGF) these cells assume many of the features of sympathetic neurons including cell cycle arrest, survival in serum-free medium, and neurite extension [17-22]. Beside NGF, which is the classical inducer of differentiation, cAMP-elevating agents, such as Pituitary Adenylate Cyclase Activating Polypeptide (PACAP), dorsomorphin and forskolin, promote growth arrest and neuritogenesis [13,23]. In NGF-free media, proteins in the extracellular matrix [24,25], electric stimulation [26] and electroactive surfaces [27] are reported to promote neurite outgrowth. In PC12 cells, the extension of neurite is one hallmark of the neuronal phenotype, along with cessation of proliferation and production of specific neurotransmitters such as nitric oxide (NO) [28]. It has been demonstrated that NGF induces NO production by the induction of all three nitric oxide synthases (NOS) isoforms [29] and that, in the absence of NGF, NO itself has the ability to produce neurite outgrowth by extracellular signal-regulated kinase (ERK) activation through NO-cGMP-PKG pathway [30].

Many authors suggest that nanotopographic guidance cues act cooperatively with NGF to regulate both the generation and the orientation of neurite even under conditions of sub-optimal NGF concentration [13-15]. Using nanostructured substrates, Ferrari *et al*. showed that in PC12 cells, stimulated by various factors including NGF, neuronal polarization and contact guidance are based on a geometrical constraint of focal adhesions and that the maintenance of the established polarity is independent from NGF stimulation while strictly dependent on the topography of the substrate [14]. Their results suggest that different neurotrophic molecules can modulate, by the selective activation of specific molecular pathways, contact guidance and the underlying establishment of cellular adhesions with the substrate. Therefore, the reading of the topographical guidance cues can be considered a function of the molecular differentiation pathway active in the cell [13].

Recently Lamour *et al.* proposed that the physical properties of the substrates can be considered as a new kind of stimulus by observing that surface free-energy gradients at the nanoscale [31,32] trigger neuritogenesis of PC12 cells in the absence of NGF or other inducers. They hypothesized that PC12 cells would respond to surface properties by secreting an unknown factor that may favor neuritogenesis, however they did not provide elements to clarify the mechanisms and the proteins involved in the physical signaling.

To address how the nanoscale stimuli distribution on a substrate is transduced into a signaling cascade, we studied the differentiation of PC12 cells on nanostructured Titania substrates fabricated by nanoparticle assembling. Our bottom-up approach, based on supersonic cluster beam deposition (SCBD) [33], offers the possibility to fabricate nanostructured TiO_2_ (ns-TiO_2_) films resulting from a random stacking of nanoparticles and characterized by a granularity and porosity mimicking those of ECM structures [34-38].

By exploiting these properties we used ns-TiO_2_ with tailored nanoscale roughness to grow PC12 in the presence and in the absence of the classical inducer of differentiation NGF in order to characterize the role of nanotopography on cell differentiation. The observed neuritogenesis triggered by the topography of ns-TiO_2_ in the absence of NGF has been studied with particular focus on the expression of NOS and the pERK1/2 signaling pathway. The human neuroblastoma SH-SY5Y cell line, which responds to retinoic acid [39], chronic NGF [40] or brain-derived neurotrophic factor (BDNF) [41], has also been used to verify if the nitration of proteins induced by nanotopography is specific for PC12 cells or can be considered a general effect in neuronal-like cell types.

## Methods

### Materials

β-mercaptoethanol, methanol, glycine, Na_2_HPO_4_, NaH_2_PO_4_, NaCl, bromophenol blue, Immobilon™-P Polyvinylidene Difluoride Membranes were purchased from Sigma-Aldrich, Inc. (Saint Louis, Missouri, USA). Nitrocellulose Membrane and Reagent Western Blot ECL Plus were obtained from GE Healthcare (Buckinghamshire, UK). Precision Plus Protein WesternC standards were purchased from Bio-Rad Laboratories, Inc. (Hercules, California, USA).

The primary antibodies used were mouse monoclonal anti-nitroTyr (32–1900) (Invitrogen, Camarillo, California, USA); mouse monoclonal anti-actin (A3853) (Sigma-Aldrich, Saint Louis, Missouri, USA); mouse monoclonal [DM1A] anti- alpha-tubulin (ab7291), rabbit polyclonal anti-NOS (ab3342), rabbit polyclonal anti iNOS (ab95441), rabbit monoclonal [EP695Y] anti-FAK (ab40794) (Abcam, Cambridge, UK); mouse p44/42 MAPK (Erk1/2) (9102) and mouse Phospho-p44/42 MAPK (Erk1/2) (Thr202/Tyr204) (Cell Signaling, Danvers, Massachusetts, USA). Referring to anti-NOS from Abcam, the antibodies utilized detect: mouse macrophage iNOS; rat, bovine, drosophila, porcine brain NOS; human, porcine, bovine eNOS.

### Substrates

Poly-L-Lysine-coated glass cover slips (64MU4113, Colaver, Milano, Italy) and microcrystalline TiO_2_ films were used as reference samples for cell culture. Flat TiO_2_ films were grown on glass slides by electron beam evaporation of a titanium target. The evaporated metal was partially oxidized during the deposition and almost fully oxidized in subsequent air exposure. To complete the oxidation and remove contaminants, these substrates were subjected to the same annealing process applied to nanostructured films, as described below.

Cluster-assembled ns-TiO_2_ substrates were grown on clean glass slides by SCBD using a Pulsed Microplasma Cluster Source (PMCS), as described in detail in [33]. Briefly, the PMCS operation principle is based on the ablation of a titanium rod by an argon plasma, ignited by a pulsed electric discharge [42]. The ablated species thermalize with the argon and condense to form clusters. The mixture of clusters and inert gas is then extracted in vacuum through an aerodynamical focusing assembly to form a seeded supersonic beam [43], the clusters are then collected on a substrate located in the beam trajectory. Since the clusters kinetic energy is low enough to avoid fragmentation, the nanoparticles impinging on the substrates maintain their original structure and, via random stacking, a nanostructured film is grown [44]. The deposition process takes place under high vacuum thus allowing the partial oxidation of the Ti clusters, further oxidation is obtained upon air exposure to atmospheric conditions and it is completed with a mild annealing for two h at the temperature of 250°C under a continuous flux of dry air. The annealing procedure has the further purpose of removing adsorbed species on the sample surfaces.

Film roughness was measured by Contact Stylus Profilometry (Dektak Veeco); the surface morphology was characterized by atomic force microscopy (AFM-Digital Instruments Nanoscope multimode IV). The AFM is equipped with rigid cantilevers with single-crystal silicon tips (nominal radius 5–10 nm) and operated in Tapping Mode. Typically, several (4–6) 2 μm × 1 μm images (2048 × 1024 points) were acquired on each sample, and flattened by line-by-line subtraction of first and second-order polynomials in order to get rid of the tilt of the sample and of the scanner bow. From flattened AFM images, the average nanoscale root-mean-square roughness and specific area parameters were calculated.

The electronic structure of as-deposited and annealed ns-TiO_2_ was characterized in a UHV (1 · 10^-9^ mbar) apparatus Leybold LHS 10/12 equipped with a hemispherical electron analyzer and conventional X-ray source (Al Kα = 1486.7 eV). The high-resolution spectra were acquired in constant pass-energy mode Epass = 30 eV with an overall energy resolution of 1.0 eV. All spectra are referenced to the Fermi level and the binding energy scale is calibrated via the Au 4f_5/2_ core level line (located at 88.5 eV) of a clean polycrystalline Au sample. No charging effects on the samples under investigation were observed during all the measurements. The line shapes were fitted with mixed singlets obtained by a linear combination of a Gaussian and a Lorentzian profiles sited on a Shirley background.

### Cell culture and analysis

#### ***Cell culture***

Rat PC12 cells (PC-12 Adh ATCC Catalog no.CRL-1721.1TM) were used as a model to test nanostructured surface effect on cell differentiation because of their faculty to assume neuronal phenotype (i.e. extension of neurites) responding to some stimuli, as (NGF). The human neuroblastoma SH-SY5Y cell line, which responds to retinoic acid, chronic NGF or BDNF, has been also used in some experiments. After annealing the glass cover slips coated with ns-TiO_2_ or flat TiO_2_ were sterilized by exposure to UV light (15 W UV lamp) for 30 min. Sterilized glass pre-coated with Poly-L-Lysine 0.01% solutions (Sigma-Aldrich) were used as positive controls.

PC12 were maintained in RPMI-1640 Medium (Sigma-Aldrich) supplemented with 10% horse serum (HS; Sigma-Aldrich), 5% fetal bovine serum (FBS; Sigma-Aldrich), 2 mM l-glutamine, 100 units/mL penicillin, 100 μg/mL streptomycin, 1 mM pyruvic acid (sodium salt) and 10 mM Hepes in 5% CO_2_, 98% air-humidified incubator (Galaxy S, RS Biotech, Irvine, California, USA) at 37°C. Cells were detached from culture dishes using a solution 1 mM EDTA in HBSS (Sigma-Aldrich), centrifuged at 1000 x g for 5 min, and resuspended in culture medium. Subcultures or culture medium exchanges were routinely established every 2^nd^ to 3^rd^ day into Petri dishes (Ø 10 cm). During the experiment the PC12 were suspended in low serum medium (1% HS) added with 50 ng/mL NGF, 2 mM S-methylisothiourea (SMT) (selective inducible NOS inhibitor) (Sigma-Aldrich), 10 μM U0126 (MEK kinase inhibitor) (Promega, Milano, Italy) and control solvent where specified, and seeded at a cell density of 5–20 × 10^4^/cm^2^ for nitration, proliferation, neurite and NOS inhibitor analysis. Following seeding, cells were maintained in 5% CO_2_, 98% air-humidified incubator at 37°C, and the medium was exchanged every 24 and 48 h after Phosphate Buffered Saline (PBS) wash. For nitration analysis, cells were seeded on rectangular glass slides (25 × 75 mm, Thermo Fisher Scientific, Milano, Italy) and cultured into 4-well rectangular dishes (Thermo Fisher Scientific). For all other analyses, cells were seeded on round cover glass (13 mm diameter, TAAB) and cultured into 24 well test plates (TPP). SH-SY5Y cells were maintained in RPM1 supplemented with 10% FCS, 1% pen/strep and 1% L-glu either on glass coverslips or nanostructured substrates, in the absence of growth factors. To label neurites, immunocytochemical staining for the protein Synaptosomal-associated protein 25 (SNAP-25) was carried out, using described methods [45].

#### ***Measurements and analysis***

Cells were imaged using an inverted phase contrast microscope (Axiovert 40 CFL; Zeiss), digital images were acquired with an AxioCam ICm1 (Zeiss) at different magnifications (Objective: LD A-Plan 20x/0.30 Ph1 and LD A-Plan 40×/0.50 Ph2) and measurements were made by ImageJ 1.44p software. The neurite length and differentiation rate were evaluated according to the following definition: the length was the straight-line distance from the tip of the neurite to the junction between the cell body and neurite base. In the case of branched neurites, the length of the longest branch was measured. For each cover glass, 20 and 40× images were acquired randomly by scanning the wells, measuring in each image: N, as total number of cells; n, as number of cells with the neurite longer than 20 μm (cells considered positive for neurite extension); l, as neurite length in μm; R, as differentiation rate determined by the equation R = 100 * n / N. Cell spreading assay: For each cover glass, 10 and 20X images were acquired randomly by scanning the wells and the cell density for cm^2^ was measured. Neurite length is presented as arithmetic mean normalized for not differentiated cell number. Each substrate type was tested 3 times with at least 100 cells considered. All data are expressed as sample arithmetic mean ± S.E.M. Significance of differences was determined using one-way ANOVA and Tukey post hoc test (* p < =0.05; ** p < =0.01).

#### ***Immunofluorescence staining***

Immunofluorescence studies were performed after 48 h from PC12 cells culture on flat TiO_2_ substrate, ns-TiO_2_ substrates (20 nm rms roughness) and PLL-glass (control). Samples were fixed and immunostained for F-actin (the actin polymer form) using an AlexaFluor555 Phalloidin probe (1:60; Life Technologies). Briefly, at room temperature cells were rinsed with PBS and fixed with 4% paraformaldehyde in PBS for 15 min; after washing, cells were permeabilized with permeabilization buffer (PBT) containing 0.2% BSA (Albumin from Bovine Serum, Fraction V, Biochemical) and 0.1% Triton X-100 for 1–5 min, blocked with 2% BSA for 1 h, stained for actin for 40 min at room temperature. Samples were rinsed twice with PBS and nuclear labeling was performed by 4′,6-diamidino-2-phenylindole (DAPI). Samples were rinsed twice with PBS, mounted with 90% glycerol and sealed. Fluorescent images were obtained with a Leica Confocal Microscopy TCS SP2.

### Lysate preparation and Western blot analysis

For preparation of whole-cell extracts, cells from cultures exposed to NGF from zero to 2 days were washed with PBS and extracted for 10 min at room temperature with sodium dodecyl sulfate polyacrylamide gel electrophoresis (SDS-PAGE) sample buffer (2% w/v SDS, 10% v/v glycerol, 5% v/v β-mercaptoethanol, 0.001% w/v bromophenol blue, and 62.5 mM Tris, pH 6.8), then the fraction was collected. To separate cytosolic and cytoskeletal-associated proteins cells were washed with PBS and extracted for 10 min at room temperature with PEM buffer (85 mM Pipes, pH 6.94, 10 mM EGTA, 1 mM MgCl_2_, 2 M glycerol, 1 mM PMSF, 0.1 mM leupeptin, 1 μM pepstatin, 2 μg/mL aprotinin) containing 0.1% v/v Triton X-100, then the fraction was collected. The obtained Triton X-100-soluble fractions were diluted 3:1 with 4X SDS-PAGE sample buffer. The insoluble material remaining attached to the dish was scraped into SDS-PAGE sample buffer. Equal proportions of each fraction, representing proteins from the same number of cells, were separated by SDS-PAGE [46].

For Western blot analysis cell lysates were resolved by SDS-PAGE, transferred to nitrocellulose or Immobilon™-P membranes, and probed with respective antibodies followed by horseradish peroxidase-conjugated secondary antibodies and detected by Enhanced Chemiluminescence method. The density of each band was estimated using the scanner GS-800 and analysis program Quantity-OneTM from BioRad Laboratories (Hercules, California, USA).

### Liquid chromatography electrospray tandem mass spectrometry (LC–ESI-MS/MS) and database analysis

For mass spectrometry analysis PC12 cell homogenates were separated by SDS-PAGE and digested *in situ* by trypsin as previously described [46]. In particular, following SDS-PAGE, each lane was cut in 2 mm bands and de-stained in 0.1% trifluoroacetic acid: acetonitrile 1:1 before drying. Gel pieces were rehydrated with trypsin (sequence grade, Sigma-Aldrich) solution (0.2 μg trypsin/band in 100 μl 50 mM ammonium bicarbonate, 9% acetonitrile), and incubated overnight at 37°C. Peptides were extracted from the gel using 0.1% trifluoroacetic acid: acetonitrile 1:1. The material was dried, resuspended in 10 μL 0.3% v/v formic acid and desalted using Zip-Tip C18 (Millipore) before mass spectrometric (MS) analysis.

Samples were separated by liquid chromatography using an UltiMate 3000 HPLC (Dionex, now Thermo Fisher Scientific). Buffer A was 0.1% v/v formic acid, 2% acetonitrile; buffer B was 0.1% formic acid in acetonitrile. Chromatography was performed using a PepMap C18 column (15 cm, 180 μm ID, 3 μm resin, Dionex). The gradient was as follows: 5% buffer B (10 min), 5%-40% B (60 min), 40%-50% B (10 min) 95%B (5 min) at a flow rate of 1.2 μL/min. Mass spectrometry was performed using a LTQ-Orbitrap Velos (Thermo Fisher Scientific) equipped with a nanospray source (Proxeon Biosystems, now Thermo Fisher Scientific). Eluted peptides were directly electrosprayed into the mass spectrometer through a standard non-coated silica tip (New Objective, Woburn, MA, USA) using a spray voltage of 2.8 kV. The LTQ-Orbitrap was operated in positive mode in data-dependent acquisition mode to automatically alternate between a full scan (m/z 350–2000) in the Orbitrap and subsequent CID MS/MS in the linear ion trap of the 20 most intense peaks from full scan. Two replicate analysis of each sample were performed. Data acquisition was controlled by Xcalibur 2.0 and Tune 2.4 software (Thermo Fisher Scientific).

Searching for nitrated proteins against the rat NCBInr database (release February 15, 2012) was performed using the Sequest search engine contained in the Proteome Discoverer 1.1 software (Thermo Fisher Scientific). The following parameters were used: 10 ppm for MS and 0.5 Da for MS/MS tolerance, carbamidomethylation of Cys as fixed modification, Met oxidation, Tyr nitration, Trp nitration and Ser/Thr/Tyr phosphorylation as variable modifications, trypsin (2 misses) as protease, False Discovery Rate for peptides 5% (against decoy), nitrated peptides identified amongst the Rank 1 peptides.

## Results and discussion

### Substrate characterization

**Figure 1:**
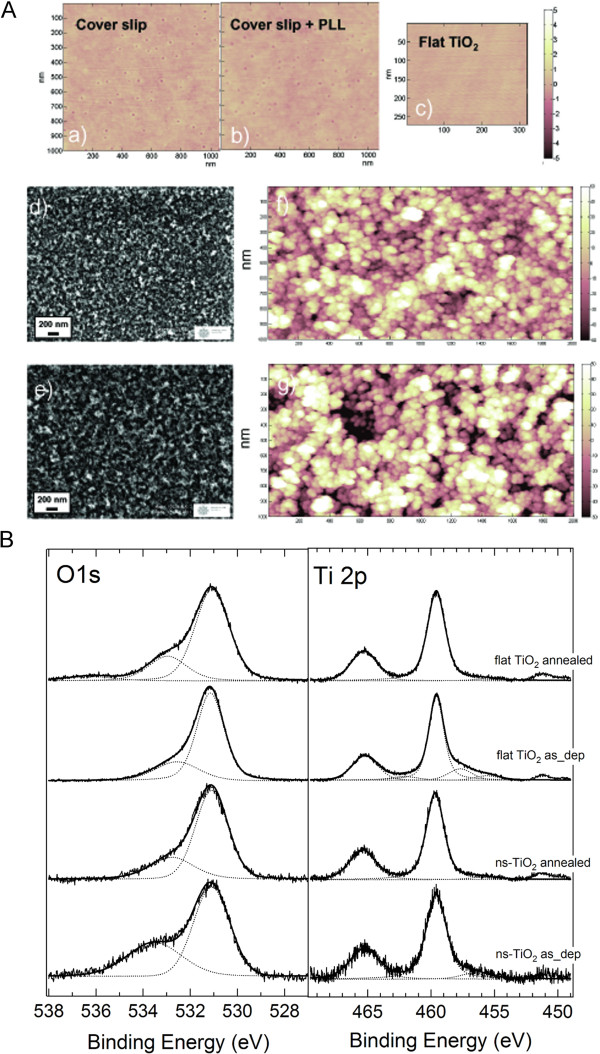
**AFM images and photoemission spectra. (A)** AFM images of reference and nanostructured samples. Reference samples: a) coverslip (1 x 1 μm^2^), rms roughness is 0.343 ± 0.004 nm; b) Poly-L-Lysine coated coverslip (1 x 1 μm^2^), rms roughness 0.271 ± 0.020 nm; c) flat TiO_2_ (2 x 1 μm^2^), rms roughness of 0.229 ± 0.004 nm. The vertical color scales range between 0 and 10 nm. Nanostructured samples: d) and e) high resolution SEM images and f) and g) AFM topographies (2 x 1 μm^2^) of 50 nm and 200 nm thick nanostructured TiO_2_, respectively. The roughness of the thinner film has been evaluated 20.2 ± 0.5 nm, whereas of the thicker film 29.1 ± 1 nm. The ns-TiO_2_ films clearly appear to have a fine raster of nanometer-sized grains with porosity at the subnanometer scale and with the thicker film showing larger height fluctuations. **(B)**. O1s and Ti 2p photoemission spectra before and after thermal annealing of flat and ns-TiO_2_ films. Ti 2p_1/2_ and Ti 2p_3/2_ peaks fall at 465.3 eV and 459.6 eV respectively (Ti(IV) bound to oxygen) and O 1 s peak shows two components one at 531.1 eV (oxygen bound to Ti(IV)) and the other at 533.5 eV (oxygen bound to contaminants).

Figure 1(A) (a)-(c) report the AFM characterization of glass and flat TiO_2_ substrates: Poly-L-Lysine coated glass has a calculated rms roughness of 0.271 ± 0.020 nm, whereas flat TiO_2_ films show a rms roughness of 0.229 ± 0.004 nm. Figure 1(d)-(g) show SEM and AFM images of cluster-assembled ns-TiO_2_ films with roughness of 20.2 ± 0.5 nm and 29.1 ± 1 nm respectively (corresponding to 50 nm and 200 nm film thickness). The random stacking of nanoparticles on substrates resulting from SCBD produces films with a homogeneous nanoscale porosity and roughness: the nanoparticles landing on the substrate stick on the surface of the growing film without any relevant diffusion or re-arrangement as it is typical of a ballistic deposition regime [36,47]. Ns-TiO_2_ substrates have been evaluated in terms of the reproducibility and control of their structural (morphology) and physico-chemical properties by accurate statistical intraslide/interslide data, showing an extremely good reproducibility among different production batches [37].

The core level photoelectron spectra at O 1 s and Ti 2p edges of nanostructured and flat TiO_2_ before and after the moderate annealing are shown in Figure 1(B). For sake of clearness the spectra of each edge have been normalized to the peak intensity. The spectra of ns-TiO_2_ appear to be noisier, attesting a larger scattering of the photoelectron emitted from the nanostructured surfaces. The peak positions of Ti 2p_1/2_ and Ti 2p_3/2_ fall at 465.3 eV and 459.6 eV respectively, corresponding to Ti(IV) bound to oxygen. The Ti 2p peaks before annealing are slightly asymmetric because of surface contamination, as OH group, which is significantly removed after thermal treatment. The FWHM of Ti2p_3/2_ is 1.8 eV, that is slightly larger than “defect-free” titanium dioxide single crystal (FWHM =1.25) as expected for ns-TiO_2_ samples having a not negligible amorphous fraction. In the O1s binding energy region, the peak at 531.1 eV (FWHM = 1.8 eV) corresponds to O 1 s core-level of oxygen atoms bound to Ti(IV), whereas the broad shoulder at higher binding energies, 533.5 eV, is mainly due to the usual oxygen sources of contaminants such as physisorbed water and carbon bounded to oxygen. The stoichiometry evaluation assesses the fully oxidation of the nanostructured and flat films.

The authors have carried out many surface characterization of the cluster assembled titanium dioxide and the effect of nanoscale roughness on film wettability and isoelectric point has been also characterized, as reported in detail in [48-50].

### TiO_2_ nanotopography triggers neuritogenesis in the absence of NGF

**Figure 2:**
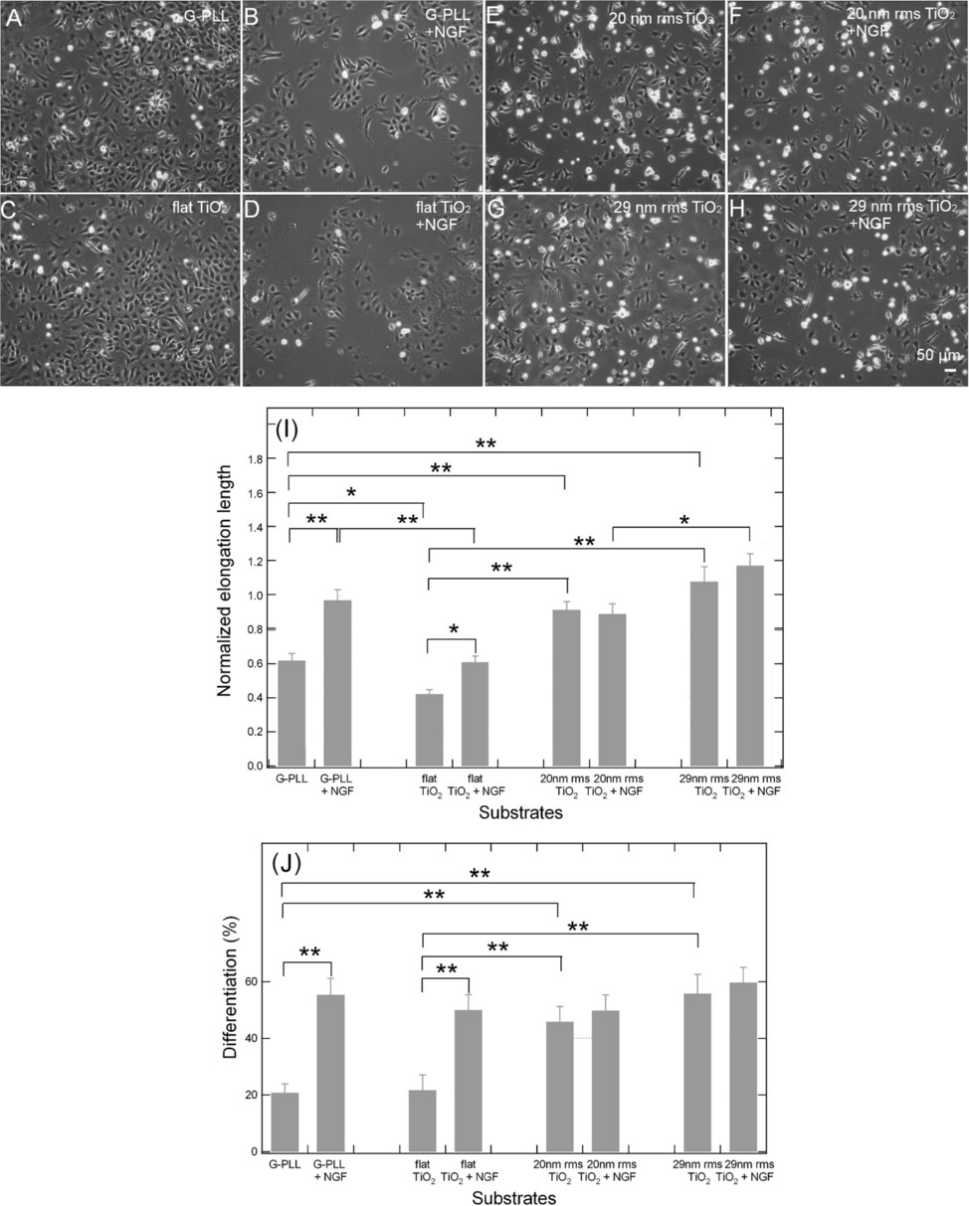
**Effect of nanostructured TiO**_**2 **_**surface on neurite formation in PC12 cells. (A-H)** Phase contrast photographs 10X magnification, Bar = 50 μm. PC12 cells were cultured for 48 h in low serum medium (1% horse serum) only **(A**, **C**, **E** and **G)** or with 50 ng/mL NGF **(B**, **D**, **F** and **H)** on four kinds of substrates: G-PLL **(A**, **B)**, flat TiO_2_**(C**, **D)**, ns-TiO_2_ 20 nm rms **(E**, **F)** and ns-TiO_2_ 29 nm rms **(G**, **H)**. **(I-L)** Histograms of the neurite length mean and differentiation percentage. Histograms of the neurite length mean **(I)** and differentiation percentage **(L)** for each condition shown in A-H. G-PLL + NGF elongation length mean and differentiation values are not statistically significantly different from ns-TiO_2_ 20 nm rms and ns-TiO_2_ 29 nm rms. * p < 0.05, ** p < 0.01, one-way ANOVA with Tukey’s post hoc test.

To test the role of the nanoscale morphology of ns-TiO_2_ in promoting neurite formation, PC12 cells were cultured on flat TiO_2_ and cluster-assembled ns-TiO_2_ substrates (20 nm and 29 nm rms roughness) either in NGF-free medium or in the presence of 50 ng/mL NGF and neurite formation was scored after 2 days (PLL-glass and flat microcrystalline TiO_2_ were used as control). Figure 2(A-H) shows phase contrast optical images with 10X magnification of PC12 cells cultured for 48 h on PLL-Glass (A) and (B), flat TiO_2_ (C) and (D), ns-TiO_2_ 20 rms (E) and (F) and ns-TiO_2_ 29 rms (G) and (H) with the following conditions: low serum medium (1% horse serum) only (A, C, E and G) or with 50 ng/mL NGF (B, D, F and H). As shown in Figure 2(E) and (G), PC12 cells cultured on ns-TiO_2_ undergo neurite expansion in NGF-free medium. After 2 days of culture neurites extend up to 103.74 μm or 154.68 μm on 20 and 29 nm rms roughness, respectively. The presence of NGF in the culture medium does not alter significantly the cell behavior: the length and number of the neurites observed are comparable between NGF-free and NGF-added medium on the same ns-TiO_2_ substrate as shown in Figure 2(I and L) where the neurite length distributions and the cell differentiation rate are reported. No significant differences in cell behavior were observed between 20 and 29 nm rms roughness ns-TiO_2_ surfaces in NGF-free medium. In contrast to the differentiation pattern observed on nanostructured Titania substrates, PC12 cells extended neurites on a PLL substrate and flat Titania only when medium was supplemented with NGF (Figure 2). Interestingly, neurite formation on PLL-glass upon NGF was equivalent to that detected on ns-TiO_2_ films in terms of both length and differentiation rate (Figure 2(L)), while cells grown on flat Titania in the presence of NGF show a similar differentiation rate but shorter elongation length (Figure 2(I)).

PC12 cells have been reported to require continuous NGF treatment for differentiation, survival and the phenotypic maintenance of the differentiated state; following cell growth longer than 2 days on ns-TiO_2_ substrates we observed that cells can survive up to 7 days on these surfaces as on glass in the presence of NGF.

**Figure 3:**
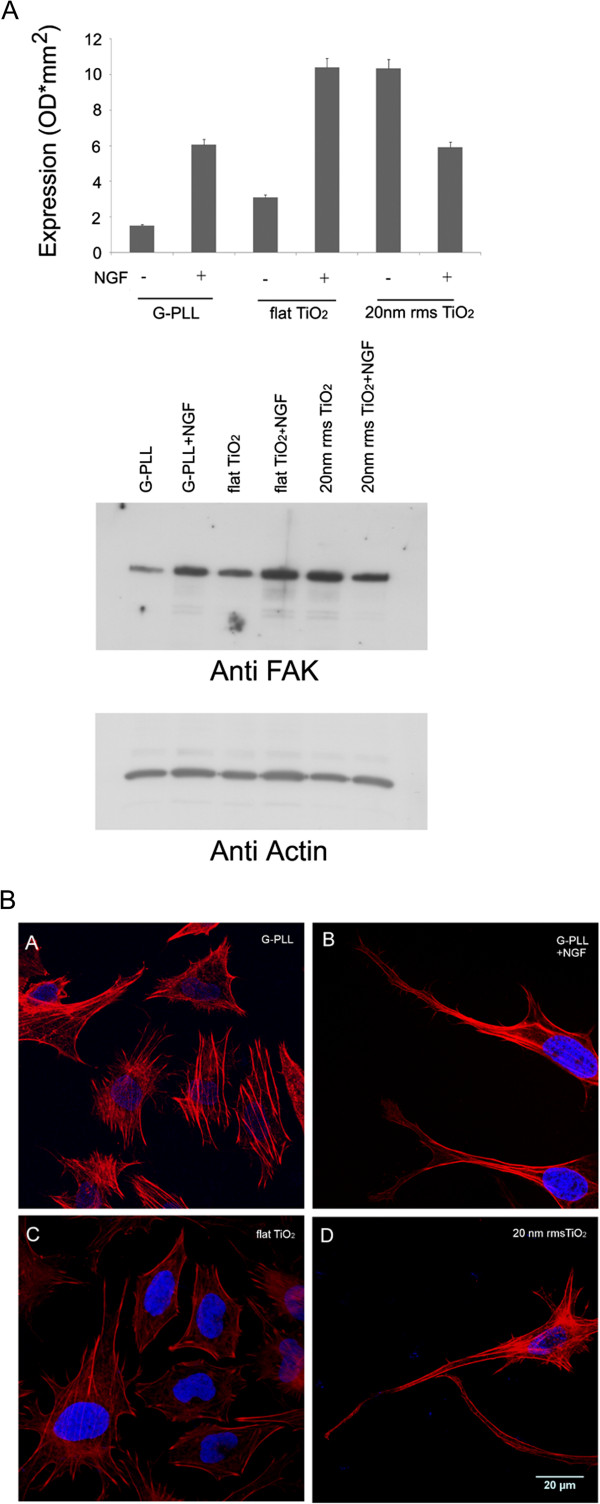
**Effect of nanotopography on FAK expression and actin cytoskeleton rearrangement. (A)** FAK expression was evaluated by Western blot using anti-FAK antibodies in PC12 cells grown on PLL-coated glass (G-PLL), flat Titania (flat TiO_2_) and nanostructured Titania (20 nm rms ns-TiO_2_) in the presence and in the absence of 50 ng/mL NGF. The results are means of 2 different experiments. **(B)**: Stress fiber organization after 48 h of culture in low serum media on different substrates and conditions: G-PLL (A), G-PLL + NGF (B), flat TiO_2_ (C) and 20 nm rms ns-TiO_2_ (D). Actin (red) and nucleus (blue) fluorescent staining.

It has been very recently demonstrated that adhesive proteins of the ECM linked with the expression of focal adhesion kinase (FAK), like collagen, fibronectin and laminin, have a profound impact on PC12 cell neurite extension [25]. On the other hand, in PC12 cells grown on biomaterials, such as highly disordered CH3/OH substrates, neuronal adhesion and differentiation mainly depend on nanoscale surface free-energy gradients [32]. To further demonstrate the correlation between nano-topography of TiO_2_ and cell differentiation, we evaluated FAK expression and actin cytoskeleton rearrangements in PC12 cells cultured on PLL-glass, on ns-TiO_2_ (20 nm rms) and on flat microcrystalline TiO_2_. As shown in Figure 3, PC12 cells seeded on ns-TiO2, without NGF treatment, underwent actin cytoskeleton reorganization associated to an increase in FAK expression. As expected, the addition of NGF leads to an increase in FAK expression also in cells seeded on PLL-Glass and on flat-TiO_2_, while the concomitant presence of two different stimuli (NGF and nano-structure) results in a decrease in FAK expression as compared to cells grown on ns-TiO_2_ without NGF, an effect that is worth investigating in more details in the future.

Compared to ref [32], our surfaces are characterized by a significant nanoroughness which has a critical influence on the observed behavior of PC12. In particular we underline the fact that protein adsorption is directly influenced by roughness at the nanoscale (as quantitatively reported and discussed in [38]), this again supporting the conclusion that the morphological cue is predominant in our system.

Altogether, these results strongly suggest that: a) nanostructure triggers neuritogenesis in the absence of other inducers, b) the phenomenon is related to the nanoscale topography of the surface, c) once triggered by surface roughness, neuritogenesis is unaffected by the addition of NGF. This implies that, in our model, topography may substitute NGF but does not act cooperatively with the chemical stimulus to promote neuritogenesis upon differentiation.

Our results are in remarkably good agreement with the observations reported by Lamour et al. using chemically modified glass surfaces as substrate for cell growth in the absence of NGF and with previous reports showing that NGF is not necessary to initiate PC12 cells differentiation [31,32].

### TiO_2_ nanotopography promotes the expression of nitric oxide synthase (NOS) and cytoskeletal proteins nitration

NO is a signaling molecule involved in NGF-induced differentiation of PC12 cells [51]. NO triggers a switch to growth arrest and neuronal differentiation [29] and it modulates neuritogenesis by regulating signaling pathways through several mechanisms [52] such as binding to heme or iron sulphur sites in regulatory proteins [53] or by modifying tyrosines in cytoskeletal proteins [37,54-56]. Unlike most other endogenous messengers that are deposited in vesicles, NO cannot be stored inside the cells, rather its signaling capacity must be controlled at the level of biosynthesis and local availability [57]. Nitric oxide synthases (NOS) are a family of enzymes which synthesize NO through the catalytic conversion of L-arginine to L-citrulline. In PC12 cells there are two forms constitutively expressed, the endothelial (eNOS) and the neuronal (nNOS), which are regulated by the cytosolic concentration of Ca^2+^[58] and an inducible isoform (iNOS) which is predominantly involved in the production of NO preceding the development of the differentiated phenotype induced by NGF [29]. The three isoforms co-localize directly or indirectly with the cytoskeleton, including actin microfilaments, microtubules and intermediate filaments [59].

**Figure 4:**
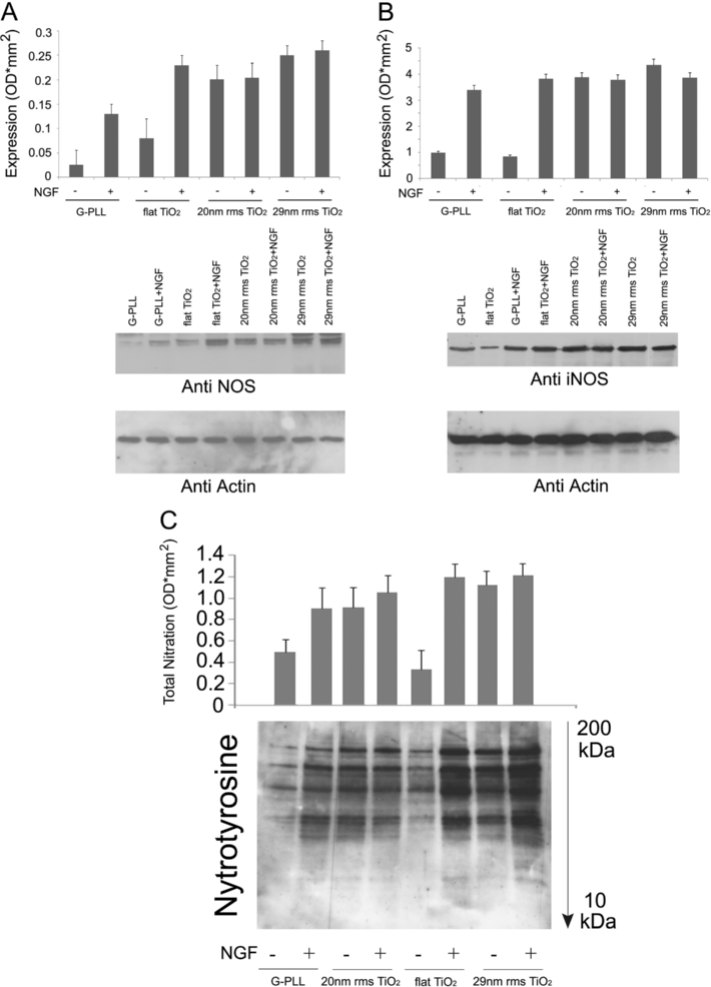
**Topography of TiO**_**2 **_**promotes the expression of nitric oxide synthase (NOS) and cytoskeletal proteins nitration.** NOS **(A)** and iNOS **(B)** expression were evaluated by Western blot analysis using anti-NOS and anti-iNOS antibodies in PC12 cells grown on PLL-coated glass (G-PLL), flat Titania (flat TiO_2_) and nanostructured Titania of different roughness (20 nm and 29 nm rms) in the presence and in the absence of 50 ng/mL NGF. **(C)** Western blot analysis using anti-nitroTyr antibodies allows to detect nitrated proteins in the Triton insoluble fraction of PC12 cells grown on PLL-coated glass (G-PLL), flat Titania (flat TiO_2_) and nanostructured Titania of different roughness (20 nm and 29 nm rms) in the presence and in the absence of 50 ng/mL NGF. Equal amounts of lysates (70 μg) were loaded on SDS-PAGE and probed with antibodies as indicated in the Methods section. The results are means of 3 different experiments.

To uncover the molecular mechanism through which nanotopography leads neuritogenesis in PC12 cells grown on ns-TiO_2_, we tested the hypothesis that NO may be involved in the process through the increase of NOS expression. This was checked by Western blot analysis using either general NOS antibodies as well as iNOS specific antibodies. The results, summarized in Figure 4(A and B), respectively, clearly show that the expression of the enzyme is increased in cells grown on nanostructured TiO_2_ similarly to the level observed on PLL-glass following NGF addition. On the contrary, cells grown on a flat TiO_2_ surface show a behavior almost overlapping the one of cells grown on PLL-glass (Figure 4(A)). These finding suggest that the morphology of the substrate modulates iNOS expression which is involved in cell differentiation as previously reported in PC12 cells grown on PLL-glass [29]. Moreover, based on the results reported in Figure 4(A) using general NOS antibodies which can detect iNOS as well as eNOS and nNOS, we do not exclude that, besides iNOS, other NOS isoforms can be involved in the process triggered by nanoscale roughness. Accordingly, it should be pointed out that, even if the expression of the constitutive NOS isoforms were not altered, their activity may be increased by nanoroughness contributing to neurite outgrowth. In this regards, recent findings clearly demonstrated that β-actin associates with eNOS and modulates NO production shifting the enzymatic activity from superoxide formation toward NO production [60].

To further confirm the involvement of NOS in the differentiation process induced by nanotopography in the absence of other stimuli, we checked for possible post-translational modifications of proteins in PC12 cells grown on ns-TiO_2_ induced by the production of NO.

In particular, we focused our attention on Tyr/Trp nitration since we previously reported that NGF triggers protein nitration during neuronal differentiation and that cytoskeleton becomes the main cellular fraction containing nitrated proteins [46].

The protein nitration was evaluated by means of anti-nitroTyr antibodies (Figure 4(C)) as well as by tandem mass spectrometry (Table 1) on the Triton insoluble fraction of PC12 cells, which is enriched in cytoskeletal components. Figure 4(C) shows that, in keeping with the results previously reported, PC12 cells grown on PLL-glass present a basal level of protein nitration which increases upon NGF induced differentiation at a level similar to the one evaluated for PC12 cells grown on ns-TiO_2_ independently from the presence of the inducer NGF. The behavior of PC12 cells grown on flat TiO_2_, on the contrary, is identical to the behavior of cells grown on PLL-glass where the increase in protein nitration is induced by NGF, thus suggesting that the nano-roughness is involved in the nitration process.

**Table 1:** List of nitrated cytoskeletal-related proteins in PC12 cells grown on the TiO2 surface (20 nm rms)

**Accession**	**Coverage**	**MW**	**pI**	**Score**	**Description**
	%				
P60711	94.67	41.7	5.48	1605.53	actin, cytoplasmic 1*
D3ZRN3	90.43	41.9	5.49	502.34	beta-actin-like protein 2
Q4V884	81.61	71.3	5.71	36.02	CDC16 cell division homolog
Q0V8T4	53.33	145.6	6.24	26.26	contactin-associated protein like 5-3
Q63164	75.47	514.7	5.90	265.33	dynein heavy chain 1, axonemal**
Q6IFW6	71.29	56.5	5.15	1004.55	keratin, type I cytoskeletal 10**
Q64715	71.12	198.4	4.84	61.81	microtubule associated protein**
P16884	83.21	115.3	5.85	25.20	neurofilament heavy polypeptide*
F1LSL8	77.51	288.6	6.02	108.33	protein Sptbn4 (spectrin beta chain, non-erythrocytic 4)**
Q9ERD7	68.00	50.4	4.93	373.64	tubulin beta-3 chain**
Q6PEC1	85.19	12.7	5.47	11.85	tubulin-specific chaperone A*

*Proteins which have been experimentally detected as nitrated also in previous studies (reviewed in [61]); **proteins for which nitration was reported for different subunits/isoforms in previous studies as compared to the present investigation [61]; all the other proteins listed in the Table are identified as nitrated for the first time in this study.

Coverage is used to determine the percent of the residues in each protein sequence that have been identified.

Score is the sum of the scores of the individual peptides belonging to a protein when analyses are performed using the Sequest search engine as indicated in the Methods section. Searching for nitrated proteins against the rat NCBInr database (release February 15, 2012) was performed using the following parameters: carbamidomethylation of Cys as fixed modification, Met oxidation, Tyr nitration, Trp nitration and Ser/Thr/Tyr phosphorylation as variable modifications, trypsin (2 misses) as protease, False Discovery Rate for peptides 5% (against decoy), nitrated peptides identified amongst the Rank 1 peptides.

**Figure 5:**
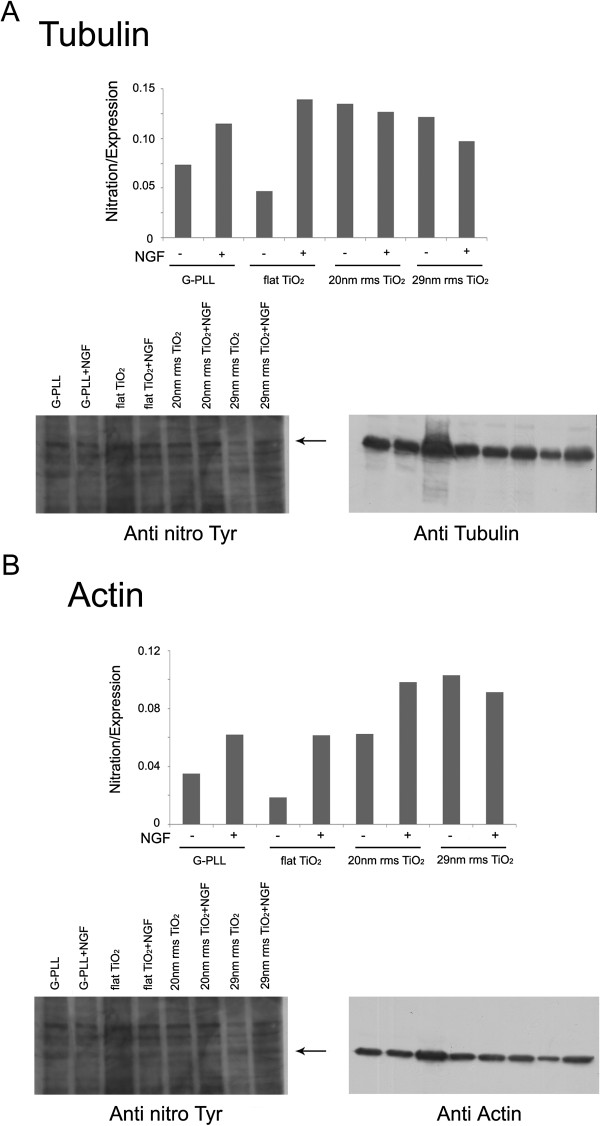
**Nitration of tubulin and actin.** The expression of tubulin **(A)** and actin **(B)** were evaluated using the corresponding specific antibodies in the Triton insoluble fraction of PC12 cells grown on PLL-coated glass (G-PLL), flat Titania (flat TiO_2_) and nanostructured Titania of different roughness (20 nm and 29 nm rms) in the presence and in the absence of 50 ng/mL NGF. Tyr nitration of the same proteins was checked following stripping of the membrane and reprobing with anti-nitroTyr antibodies as described in the Methods section. The results are means of 2 different experiments.

The identification of the proteins found nitrated in PC12 cells grown on different TiO_2_ substrates in NGF free media was carried out by tandem mass spectrometry looking for peptides containing at least one nitration at Tyr and/or Trp residues. In keeping with the previous findings [46], many of them are components of the cytoskeleton as shown in Table 1, which reports the list of the cytoskeletal proteins found nitrated in such conditions. As reported in [46,62] alpha-tubulin, and actin are among the major target of this post-translational modification which may confer increase stability to cytoskeleton during neuronal differentiation [63]. Therefore, the expression of tubulin and actin were specifically evaluated using the corresponding antibodies while their Tyr nitration was checked following stripping of the membrane and reprobing with anti-nitroTyr antibodies. The results are summarized in Figure 5 where the ratio between nitration and expression is reported for each protein tested. The pattern of their nitration follows the same pattern reported above for protein nitration in general (Figure 4(C)) confirming that the nanoscale roughness induces nitration in the absence of NGF.

### Effect of NOS inhibitor on PC12 cells grown on nanostructured TiO_2_

**Figure 6:**
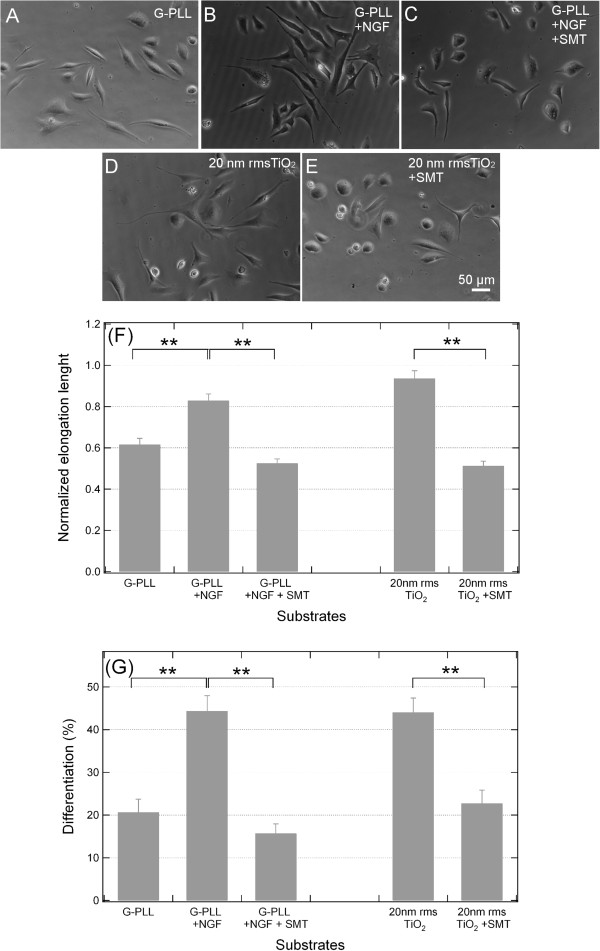
**Effect of iNOS inhibitors on neurite formation in PC12 cells induced by nanostructured TiO**_**2 **_**surface. (A-E)**: Phase contrast photographs 20X magnification, Bar = 50 μm. PC12 cells were cultured on PLL-coated coverslips for 48 h in low serum medium (1% horse serum) only **(A)**, with 50 ng/mL NGF **(B)** or with 50 ng/mL NGF and 2 mM SMT **(C)**. PC12 cells were cultured on ns-TiO_2_ (20 nm rms) for 48 h in low serum medium (1% horse serum) only **(D)** or with 2 mM SMT **(E)**. **(F**,**G)**: Histograms of the neurite length mean and differentiation percentage in the presence of iNOS inhibitor. Histograms of the neurite length mean **(F)** and differentiation percentage **(G)** in the presence of iNOS inhibitor (2 mM SMT) for each condition shown in A-E. ** p < 0.01, one-way ANOVA with Tukey’s post hoc test.

To ascertain that NOS is critical in PC12 cell differentiation triggered by the substrate nanostructure, cells were grown in the presence of NOS inhibitor SMT. As shown in Figure 6, PC12 cells cultured under control conditions on PLL-glass undergo neurites expansion and differentiation only in the presence of NGF and both processes are hampered by incubation with SMT. The same effect was observed when PC12 cells were cultured on ns-TiO_2_ of 20 nm rms roughness in NGF-free medium: Figure 6(D-G) clearly show that prevention of neurite growth and differentiation is induced by SMT also under this growing condition at an extent similar to the one observed on PLL-glass. Altogether, these results clearly suggest that NOS is involved in cell differentiation observed in PC12 cells grown on ns-TiO_2_ without NGF. In particular, since iNOS has been described as the enzyme predominantly involved in the production of NO preceding the development of the differentiated phenotype induced by NGF in PC12 cells grown on PLL-glass, the results suggest that iNOS is involved in the differentiation process also in our experimental system. This is in keeping with the data of NOS expression reported in Figure 4 and confirms our hypothesis that nanotopography mimics the effect of NGF, promoting NOS expression and cytoskeletal protein nitration.

### Effect of nanostructured TiO_2_ on the human neuroblastoma SH-SY5Y cell line

**Figure 7:**
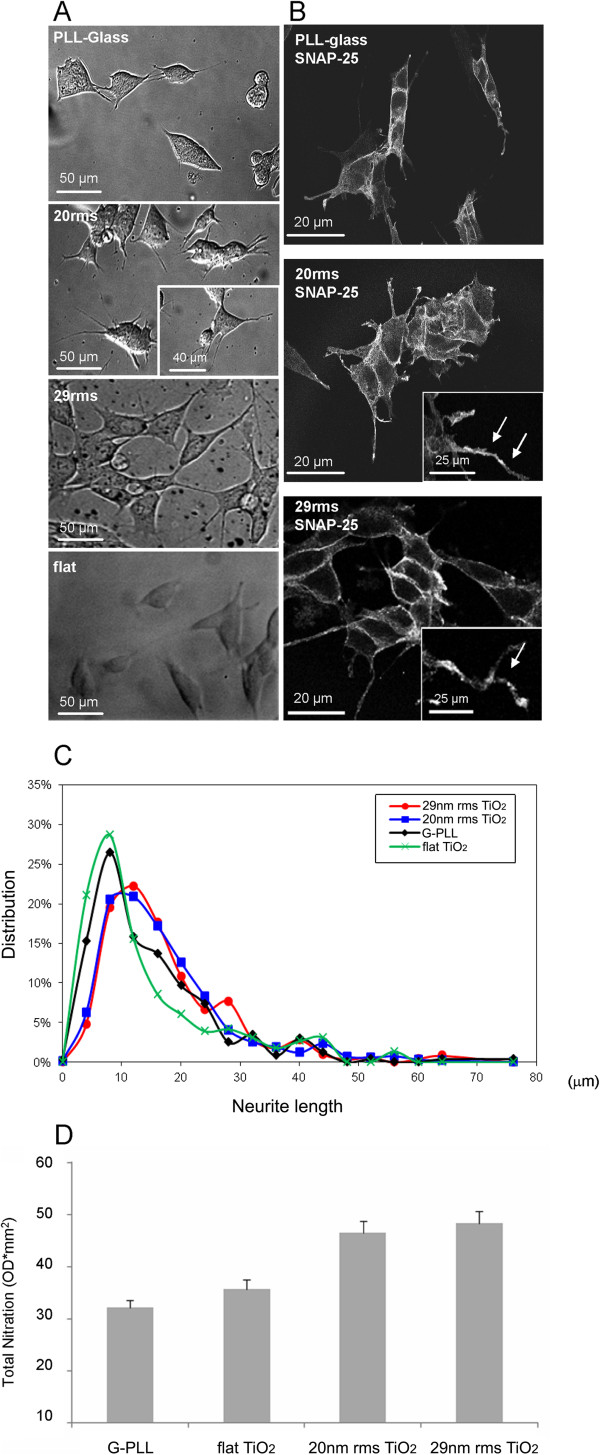
**Ns-TiO**_**2 **_**surface nanotopography increases neurite length in human neuroblastoma cell line and triggers protein nitration. (A)** bright field and SNAP-25 immunostaining of SY5Y cells plated on glass, TiO_2_-flat and nanostructured Titania (TiO_2_ 20 nm rms and TiO_2_ 29 nm rms); **(B)** bright field and SNAP-25 immunostaining of SY5Y cells plated on ns-TiO_2_. Insets show details of neurites. **(C)** neurite length distribution of cells grown on glass (G-PLL), flat TiO_2_ or on ns-TiO_2_. Kolmogorov-Smirnov: G-PLL vs. TiO_2_ 20 nm rms (p < 0.001), glass vs. TiO_2_ 29 nm rms (p < 0.001), TiO_2_ 20 nm rms vs. TiO_2_ 29 nm rms not significantly different, G-PLL vs. flat TiO_2_ not significantly different. **(D)** Histogram shows Western blot analysis using anti-nitroTyr antibodies. The results are means of 2 different experiments.

We then aimed at defining whether the effects produced by nanostructured TiO_2_ on neurite growth was specific for PC12 cells or was a generalized effect produced by the substrate on different neuronal-like cell types. Therefore, we studied the behaviour on glass or ns-TiO_2_ 20 nm and 29 nm rms roughness of SH-SY5Y human neuroblastoma cells which are considered as in vitro cell model of dopaminergic neurons and have been widely studied as cell model for Parkinson’s disease (reviewed in [64]). As shown in the case of PC12 cells, neuroblastoma cells grown on 20 or 29 nm rms ns-TiO_2_ displayed longer neuritis with respect to cells grown on glass or on flat substrates, as revealed by bright field examination (Figure 7A), as well as by the staining for the protein SNAP-25 (Figure 7B) [65]. The neurite length distributions analysis showed an evident shift of the normal distribution toward higher length values. No difference between different ns-TiO_2_ roughnesses was observed (Figure 7A and C). Western blot analysis by anti-nitroTyr antibodies, shows that there is an increase in protein nitration triggered by the ns-TiO_2_ as described above in PC12 cells (Figure 7D) suggesting that this behavior is common to different neuronal-like cell types. Interestingly, in SY5Y cells evidence in literature indicates that marked increases in the levels of nitrated proteins induce apoptotic cell death [64]. We show here that modest induction of protein nitration induces instead increased neuritogenesis in the same cell line.

### Involvement of ERK signaling cascade in nanostructured-induced neuritogenesis

**Figure 8:**
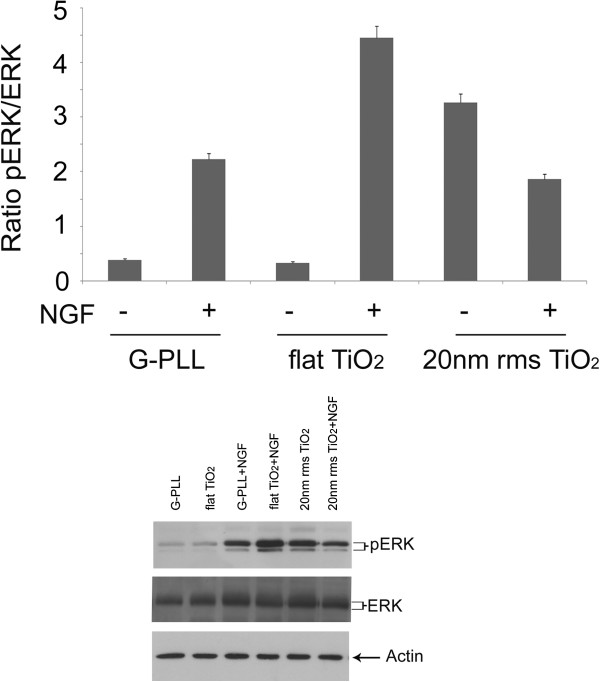
**Involvement of ERK signaling cascade in nanostructured-induced neuritogenesis.** The expression and the phosphorylation of ERK were evaluated by Western blot analysis using anti-ERK and anti-p-ERK antibodies, on PC12 cells grown on PLL-coated glass (G-PLL), flat Titania (flat TiO_2_) and nanostructured Titania (20 nm rms TiO_2_) in the presence and in the absence of 50 ng/mL NGF. Equal amounts of lysates (70 μg) were loaded on SDS-PAGE and probed with antibodies as indicated in the Methods section. The results are means of 3 different experiments.

The addition of NGF to PC12 cells causes neurite elongation through a sustained activation of ERK, a mitogen-activated protein kinase whose phosphorylation is essential to neuronal differentiation [66]. As reported by Yamazaki et al. [30], this activation occurs upon activation of NOS and can be obtained also by NO itself, in the absence of NGF, during NO-induced neuritogenesis. These observations prompted us to check if the ERK-signaling cascade may be also involved in the differentiation process triggered by nanotopography. We checked the expression of ERK and its phosphorylation by Western blot analysis using anti-ERK and anti-p-ERK antibodies. The results, summarized in Figure 8, clearly show that when cells are grown on ns-TiO_2_ in NGF-free media ERK is phosphorylated to the same extent as in cell grown on glass or on flat TiO_2_ upon stimulation by NGF. In the latter two substrates the activation of ERK is almost undetectable in the absence of NGF.

**Figure 9:**
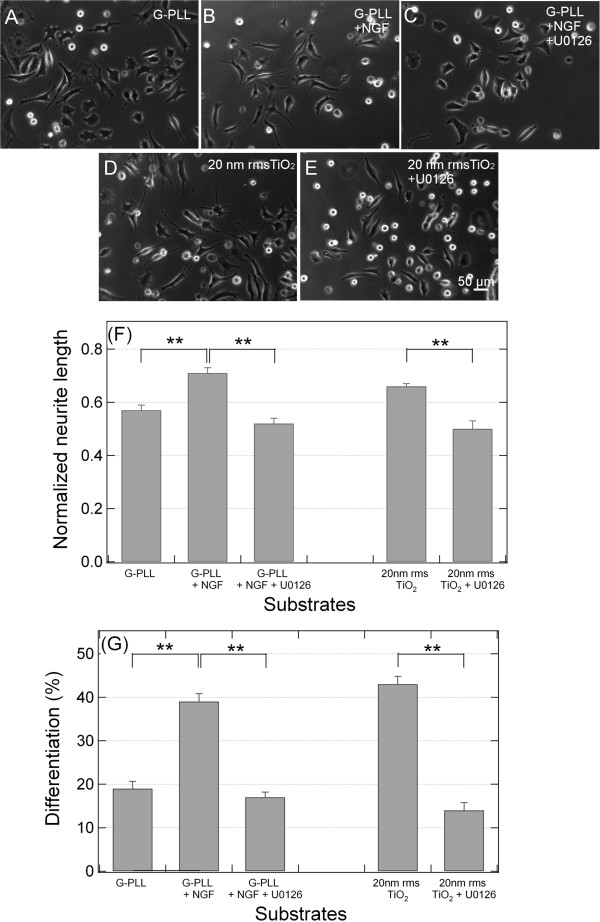
**Inhibitory effect of the MEK kinase inhibitor U0126 on PC12 neurite extension induced by TiO2 nanostructure. (A-E)**: Phase contrast photographs 20X magnification, Bar = 50 μm PC12 cells were cultured on PLL-coated coverslips for 48 h in low serum medium (1% horse serum) only **(A)**, with 50 ng/mL NGF **(B)** or with 50 ng/mL NGF and U0126 **(C)**. PC12 cells were cultured on ns-TiO_2_ (20 nm rms) for 48 h in low serum medium (1% horse serum) only **(D)** or with U0126 **(E)**. In C and E the MEK kinase inhibitor U0126 was added to a final concentration of 10 μM. **(F-G)**. Histograms of the neurite length mean and the differentiation percentage relative to Figure 9**(A-E)**. Histograms showing the neurite length mean **(F)** and the differentiation percentage **(G)** in the presence of the MEK kinase inhibitor (10 μM U0126) for each condition shown in A-E. Cells treated with the inhibitor display a significant suppression of neurite outgrowth compared to controls. ** p < 0.01, one-way ANOVA with Tukey’s post hoc test.

To further confirm the involvement of the ERK signaling cascade in the process, we tested the effect of an inhibitor of MEK kinase, the enzyme responsible for ERK activation in the signaling cascade. As shown in Figure 9, cells treated with the inhibitor display a significant suppression of neurite outgrowth compared to control conditions, both on PLL plus NGF and on ns-TiO_2_, and present a behavior similar to unstimulated cells (PLL-glass only). Accordingly, differentiation induced by NGF on PLL-glass and by ns-TiO_2_ is prevented by MEK kinase inhibitor to a similar extent, suggesting that the same pathway is involved in differentiation process started by the two different stimuli.

Our data are in extremely good agreement with previous findings by Foley et al. [15] who described the involvement of ERK in the differentiation of PC12 cells cultured on synthetic substrates whose topographical features act to modulate neuritogenesis under sub-optimal concentration of NGF. Since NGF treatment has been shown to up-regulate α_1_β_1_ integrin molecules in PC12 cells [67,68] and integrin-mediated FAK activation augments EGF/ERK signaling [69], they suggested that the formation and organization of focal adhesions on nanoscale features may cooperate with NGF to promote neuritogenesis when the concentration of the chemical inducer is low while it is ineffective at 50 ng/mL NGF when the signaling cascade is already at its maximum. This is in accordance with our finding that nanotopography mimics the effect of NGF but it does not act cooperatively with NGF to promote neuritogenesis. Based on our finding, we propose that the perturbation of the actin cytoskeleton caused by the surface nanoroughness, shown in the immunostaining results reported in Figure 3B(A-D), increases NOS expression and the NO-signaling cascade activation as well as ERK activation therefore explaining the cell behavior observed on nanostructured TiO_2_.

One question arises from this picture: how nanotopography may increase NOS expression in order to produce NO. Many data suggest that NOS activity may be regulated by cytoskeleton at transcriptional, post-transcriptional and post-translational level [59] and that the cytoskeletal reorganization induced by extracellular stimuli such as shear stress, hypoxia and drugs play an important role in regulating NOS expression and activity [59,70]. iNOS gene transcription is regulated by changes in the actin cytoskeleton in alveolar epithelial cells [71], glomerular mesangial cells [72] and vascular smooth muscle cells [73,74]. In macrophages it is proposed that microtubule depolymerisation activates stress fibers formation through regulation of iNOS gene expression by actin microfilaments [75-77]. Moreover, in these cells the interaction of iNOS with actin binding protein α-actinin has been demonstrated [59]. Co-localization of nNOS with cytoskeleton in skeletal muscle cells optimizes NO production, improving metabolism, elasticity and mechanical properties of the cells [78,79]. Recently, Gupta et al. [80] demonstrated a clear interaction between integrins and iNOS in modulation of cell migration. Their results clearly show that integrin α_9_β_1_ enhances cell migration through production of NO by iNOS regulated by SRC tyrosine kinase [80]. Moreover, the iNOS/SRC/FAK axis was found to be critical in cell mobility processes in macrophages [81]. Based on all these observations it is possible to speculate that in the differentiation of PC12 cells triggered by nanostructure the cytoskeletal rearrangements may lead to an increase in NOS expression, NO production and modulation of ERK signaling, similarly to what recently reported by Miyamoto et al. who described that nNOS expression enhances ERKs phosphorylation in nNOS.transfected PC12 cells [82]. Modulation of the MAK kinase pathway in PC12 by NO/NOS has been described by several laboratories [30,83,84] suggesting that NOS induction/activation is upstream to the MAPK cascade in the signaling process of neuritogenesis. On the other hand, numerous papers provided evidence that the ERK pathway is required for the induction of nNOS in NGF-differentiated PC12 cells [85,86], in rat aortic smooth muscle cells [87] and in an experimental model of brain stem death in rat rostral ventrolateral medulla [88], while other evidences describe the role played by the MAP kinase pathway in regulating the expression and the phosphorylation state of eNOS [89-91]. Moreover, Cragg et al. proposed, very recently, a model in which NO is involved in the prolonged activation of MAP kinase which then contributes to the NGF-mediated increase in eNOS and iNOS mRNA levels rather than nNOS expression [92]. In summary, these and other findings suggest that a very complex, and still partially undiscovered, reciprocal modulation amongst cytoskeletal proteins, NOS and MAP kinase pathway (as far as expression levels, post translational modifications and biological activity are concerned) is involved in several processes, including NGF-induced differentiation of PC12. The present report suggests that the same molecular players are involved also in the differentiation induced by surface topography of nanostructured TiO_2_. More experimental data are required to exactly enlighten the mechanism underlying the differentiation induced either by NGF or by nano-roughness, including investigations concerning the possibility that cytoskeletal changes may increase eNOS activity and NO production which can then be involved in ERK phosphorylation together with induction of one or more NOS isoforms. Furthermore, our data suggest that nitration of cytoskeletal proteins may be one additional critical mechanism active in cell differentiation.

## Conclusions

We studied the behavior of PC12 cells on ns-TiO2 films in the presence and in the absence of the inducer of differentiation NGF. We showed that, in PC12 cells grown in the absence of NGF, the nanotopography of ns-TiO2 triggers neuritogenesis by stimulating the expression of NOS and the pERK1/2 signaling pathway. By comparing Titania surfaces with different roughness at the nanoscale we demonstrated that the observed behavior is not affected by the chemistry but only by the topography of the substrates. Differentiation is associated to an increase in protein nitration as observed in PC12 cells grown on PLL-glass in the presence of NGF.

Altogether our data show for the first time that the NO signal cascade is involved in the differentiation process induced by nanotopography, adding new information on the mechanism and proteins involved in the neuritogenesis process triggered by the surface properties and suggesting that NO could be the “unknown” factor produced by PC12 cells in response to surface properties that Lamour *et al*. recently proposed in order to explain the influence of nanoscale surface energy distribution on neuritogenesis [31,32].

As in the case of nanoscale chemical inhomogeneities [31,32], our results define the role of nanoscale morphology as a biomaterial design parameter to dissect the molecular pathways related to cell adhesion and differentiation showing that the morphological parameter regulating the NOS pathway is the nanoscale morphology. By comparing Titania surfaces with different roughness we demonstrated that the observed behavior is affected by the nanoscale topography of the substrates which is dictating the signaling cascade originating from the modulation of culture media proteins adhering on the substrates.

This finding is highly significant for many applications where nanostructures interact with biological systems, for the understanding of cell-nanostructured surface interaction and for the general understanding of the nano-bio interface. In particular the use of surfaces with controlled and reproducible roughness at the nanoscale, as ns-TiO_2_, will allow addressing a major issue concerning the physiological role played by NO through nitration of cytoskeletal proteins in many cytoskeleton-mediated processes such as cell growth and division.

## Abbreviations

NGF: Nerve growth factor; NO: Nitric oxide; NOS: Nitric oxide synthase; iNOS: Inducible nitric oxide synthase; eNOS: Endothelial nitric oxide synthase; nNOS: Neuronal nitric oxide synthase; ERK 1/2: Extracellular signal-regulated kinase 1/2; ECM: Extracellular matrix; PACAP: Pituitary adenylate cyclase activating polypeptide; SCBD: Supersonic cluster beam deposition; BDNF: Brain-derived neurotrophic factor; MAP kinase: Mitogen-activated kinase; FAK: Focal adhesion kinase; G-PLL: Poly-L-lysine-coated glass; ns-TiO2: Nanostructured titanium oxide; PMCS: Pulsed microplasma cluster source; AFM: Atomic force microscopy; SMT: S-methylisothiourea; MEK: Mitogen-activatd kinase/extracellular signal-regulated kinase kinase; PBS: Phosphate buffered saline; SNAP-25: Synaptosomal-associated protein 25; CID MS/MS: Collision induced dissociation tandem mass spectrometry; rms: Root mean square; SEM: Scanning electron microscope; EGF: Epidermal growth factor; SRC: Proto-oncogene tyrosine-protein kinase Src; DAPI: 4′,6-diamidino-2-phenylindole; PKG: Protein kinase G.

## Competing interests

The authors declare that they have no competing interests.

## Authors’ contributions

MT carried out all the experiments of TiO_2_ substrate preparation, substrate characterization, PC12 cells growth and helped to draft the manuscript; CL, SF and ES participated in substrate preparation and characterization; EM and SN carried out Western blot experiments; AN participated in the mass spectrometry analysis; SDA and MM carried out the experiments concerning the human neuroblastoma SH-SY5Y cell line; CS participated in PC12 cells growth and imaging analysis; PM participated in the design of the study, supervised all the experiments of TiO_2_ substrate preparation and characterization and helped to draft the manuscript; GT was involved in conceiving the study, supervised the experiments design to test protein nitration and drafted the manuscript. All authors read and approved the final manuscript.

## References

[B1] Lutolf MP Hubbell JA Synthetic biomaterials as instructive extracellular microenvironments for morphogenesis in tissue engineering Nat Biotechnol 2005 23 47 55 10.1038/nbt1055 15637621

[B2] Kleinman HK Philp D Hoffman MP Role of the extracellular matrix in morphogenesis Curr Opin Biotechnol 2003 14 526 532 10.1016/j.copbio.2003.08.002 14580584

[B3] Chen C Jiang X Microengineering the environment of mammalian cells in culture MRS Bull 2005 30 194 201 10.1557/mrs2005.52

[B4] Wheeldon I Farhadi A Bick AG Jabbari E Khademhosseini A Nanoscale tissue engineering: spatial control over cell-materials interactions Nanotechnology 2011 22 212001 10.1088/0957-4484/22/21/212001 21451238 PMC3155808

[B5] Abrams GA Goodman SL Nealey PF Franco M Murphy CJ Nanoscale topography of the basement membrane underlying the corneal epithelium of the rhesus macaque Cell Tissue Res 2000 299 39 46 10.1007/s004410050004 10654068

[B6] Dalby MJ Riehle MO Johnstone H Affrossman S Curtis AS In vitro reaction of endothelial cells to polymer demixed nanotopography Biomaterials 2002 23 2945 2954 10.1016/S0142-9612(01)00424-0 12069336

[B7] Andersson AS Backhed F Von Euler A Richter-Dahlfors A Sutherland D Kasemo B Nanoscale features influence epithelial cell morphology and cytokine production Biomaterials 2003 24 3427 3436 10.1016/S0142-9612(03)00208-4 12809771

[B8] Thapa A Webster TJ Haberstroh KM Polymers with nano-dimensional surface features enhance bladder smooth muscle cell adhesion J Biomed Mater Res 2003 67 1374 1383 10.1002/jbm.a.20037 14624525

[B9] Dalby MJ Gadegaard N Riehle MO Wilkinson CD Curtis AS Investigating filopodia sensing using arrays of defined nano-pits down to 35 nm diameter in size Int J Biochem Cell Biol 2004 36 2015 2025 10.1016/j.biocel.2004.03.001 15203114

[B10] Dalby MJ Riehle MO Johnstone HJ Affrossman S Curtis AS Polymer-demixed nanotopography: control of fibroblast spreading and proliferation Tissue Eng 2002 8 1099 1108 10.1089/107632702320934191 12542955

[B11] Yim EKF Darling EM Kulangara K Guilak F Leong KW Nanotopography-induced changes in focal adhesions, cytoskeletal organization, and mechanical properties of human mesenchymal stem cells Biomaterials 2010 31 1299 1306 10.1016/j.biomaterials.2009.10.037 19879643 PMC2813896

[B12] Fan YW Cui FZ Hou S Xu QY Chen LN Lee IS Culture of neural cells on silicon wafers with nano-scale surface topograph J Neurosci Methods 2002 120 17 23 10.1016/S0165-0270(02)00181-4 12351203

[B13] Ferrari A Faraci P Cecchini M Beltram F The effect of alternative neuronal differentiation pathways on PC12 cell adhesion and neurite alignment to nanogratings Biomaterials 2010 31 2565 2573 10.1016/j.biomaterials.2009.12.010 20035995

[B14] Ferrari A Cecchini M Serresi M Faraci P Pisignano D Beltram F Neuronal polarity selection by topography-induced focal adhesion control Biomaterials 2010 31 4682 4694 10.1016/j.biomaterials.2010.02.032 20304485

[B15] Foley AD Grunwald EW Nealey PF Murphy CJ Cooperative modulation of neuritogenesis by PC12 cells by topography and nerve growth factor Biomaterials 2005 26 3639 3644 10.1016/j.biomaterials.2004.09.048 15621254

[B16] Schwarz US Bischofs IB Physical determinants of cell organization in soft media Med Eng Phys 2005 27 763 772 10.1016/j.medengphy.2005.04.007 15951217

[B17] Huang C Borchers CH Schaller MD Jacobson K Phosphorylation of paxillin by p38MAPK is involved in the neurite extension of PC-12 cells J Cell Biol 2004 26 593 602 14970194 10.1083/jcb.200307081 PMC2171993

[B18] Woo S Gomez TM Rac1 and RhoA promote neurite outgrowth through formation and stabilization of growth come point contacts J Neurosci 2006 27 730 742 10.1523/JNEUROSCI.4209-05.2006 PMC6675502 16452665

[B19] D’Arcandelo G Halegoua S A branched signaling pathway for nerve growth factor is revealed by Src-, Ras-, and Raf-mediated gene inductions Mol Cell Biol 1993 13 3146 3155 8497245 10.1128/mcb.13.6.3146-3155.1993 PMC359751

[B20] Klesse LJ Meyers KA Marshall CJ Para LF Nerve growth factor induces survival and differentiation through two distinct signaling cascades in PC12 cells Oncogene 1999 18 2055 2068 10.1038/sj.onc.1202524 10321730

[B21] Rakhit S Pyne S Pyne NJ Nerve growth factor stimulation of p42/p44 mitogen activated protein kinase in PC12 cells: Role of G(i/o), G protein –coupled receptor kinase 2, beta-arrestin I, and endocytic processing Mol Pharmacol 2001 60 63 70 11408601 10.1124/mol.60.1.63

[B22] Waetzig V Herdegen T The concerted signaling of ERK1/2 and JNKs is essential for PC12 cell neuritogenesis and converges at the level of target proteins Mol Cell Neurosci 2003 24 238 249 10.1016/S1044-7431(03)00126-X 14550783

[B23] Greene LA Tischler AS Establishment of noradrenergic clonal line of rat adrenal pheochromocytoma cells which respond to nerve growth factor Proc Natl Acad Sci USA 1976 73 2424 2428 10.1073/pnas.73.7.2424 1065897 PMC430592

[B24] Fujii DK Massoglia SL Savion N Gospodarowicz D Neurite outgrowth and protein synthesis by PC12 cells as a function of substratum and nerve growth factor J Neurosci 1982 2 1157 1175 7108587 10.1523/JNEUROSCI.02-08-01157.1982 PMC6564279

[B25] Lee JH Lee HY Kim HW Adhesive proteins linked with focal adhesion kinase regulate neurite outgrowth of PC12 cells Acta Biomater 2012 8 165 172 10.1016/j.actbio.2011.08.024 21911085

[B26] Aizawa M Koyama S Kimura K Haruyama T Yanagida Y Kobatake E Electrically stimulated modulation of cellular function in proliferation, differentiation, and gene expression Electrochemistry 1999 67 118 125

[B27] Guo Y Li M Mylonakis A Han J MacDiarmid AG Chen X Electroactive oligoaniline-containing self-assembled monolayers for tissue engineering applications Biomacromolecules 2007 8 3025 3034 10.1021/bm070266z 17845053

[B28] Gerdin MJ Eiden LE Regulation of PC12 cell differentiation by cAMP signaling to ERK independent of PKA: do all the connections add up? Sci STKE 2007 382 pe15 17440132 10.1126/stke.3822007pe15 PMC4183209

[B29] Peunova N Enikolopov G Nitric oxide triggers a switch to growth arrest during differentiation of neuronal cells Nature 1995 375 68 73 10.1038/375068a0 7536899

[B30] Yamazaki M Chiba K Mohri T Fundamental role of nitric oxide in neuritogenesis of PC12h cells Br J Pharmacol 2005 146 662 669 10.1038/sj.bjp.0706370 16113690 PMC1751193

[B31] Lamour G Journiac N Souès S Bonneau S Nassoy P Hamraoui A Influence of surface energy distribution on neuritogenesis Colloids Surf B: Biointerfaces 2009 72 208 218 10.1016/j.colsurfb.2009.04.006 19419846

[B32] Lamour G Aftekhari-Bafrooei A Borguet E Souès A Hamraoui A Neuronal adhesion and differentiation driven by nanoscale surface free-energy gradients Biomaterials 2010 31 3762 3771 10.1016/j.biomaterials.2010.01.099 20149439

[B33] Wegner K Piseri P Vahedi Tafreshi H Milani P Cluster beam deposition: a tool for nanoscale science and technology J Phys D Appl Phys 2006 39 R439 R459 10.1088/0022-3727/39/22/R02

[B34] Carbone E Marangi I Zanardi A Giorgetti L Chierici E Berlanda G Biocompatibility of cluster-assembled nanostructured TiO_2_ with primary and cancer cells Biomaterials 2006 27 3221 3229 10.1016/j.biomaterials.2006.01.056 16504283

[B35] Bellicchi M Erratico S Razini P Meregalli M Cattaneo A Jacchetti E Ex vivo expansion of human circulating myogenic progenitors on cluster-assembled nanostructured TiO_2_ Biomaterials 2010 31 5385 5396 10.1016/j.biomaterials.2010.03.021 20398929

[B36] Podestà A Bongiorno G Scopelliti PE Bovio S Milani P Semprebon G Cluster-assembled nanostructured titanium oxide films with tailored wettability J Phys Chem C 2009 113 18264 18269 10.1021/jp905930r

[B37] Carbone R De Marni M Zanardi A Vinati S Barborini E Fornasari L Characterization of cluster-assembled nanostructured titanium oxide coatings as substrates for protein arrays Anal Biochem 2009 394 7 12 10.1016/j.ab.2009.07.005 19589333

[B38] Scopelliti PE Borgonovo A Indrieri M Giorgetti L Bongiorno G Carbone R The effect of surface nanometre-scale morphology on protein adsorption PLoS ONE 2010 5 e11862 10.1371/journal.pone.0011862 20686681 PMC2912332

[B39] Påhlman S Ruusala AI Abrahamsson L Mattsson ME Esscher T Retinoic acid-induced differentiation of cultured human neuroblastoma cells: a comparison with phorbolester-induced differentiation Cell Differ 1984 14 2 135 144 10.1016/0045-6039(84)90038-1 6467378

[B40] Jensen LM Zhang Y Shooter EM Steady-state polypeptide modulations associated with nerve growth factor (NGF)-induced terminal differentiation and NGF deprivation-induced apoptosis in human neuroblastoma cells J Biol Chem 1992 267 27 19325 19333 1527053

[B41] Jämsä A Hasslund K Cowburn RF Bäckström A Vasänge M The retinoic acid and brain-derived neurotrophic factor differentiated SH-SY5Y cell line as a model for Alzheimer’s disease-like tau phosphorylation Biochem Biophys Res Commun 2004 319 3 993 1000 10.1016/j.bbrc.2004.05.075 15184080

[B42] Vahedi Tafreshi H Piseri P Benedek G Milani P The role of gas dynamics in operation conditions of a pulsed microplasma cluster source for nanostructured thin films deposition J Nanosci Nanotechnol 2006 6 1140 1149 10.1166/jnn.2006.139 16736779

[B43] Piseri P Vahedi Tafreshi H Milani P Manipulation of nanoparticles in supersonic beams for the production of nanostructured materials Curr Opin Solid State Mater Sci 2004 8 195 202 10.1016/j.cossms.2004.08.002

[B44] Barborini E Kholmanov IN Conti AM Piseri P Vinati S Milani P Supersonic cluster beam deposition of nanostructured titania Eur Phys J D 2003 24 277 282

[B45] Verderio C Coco S Fumagalli G Matteoli M Spatial changes in calcium signaling during the establishment of neuronal polarity and synaptogenesis J Cell Biol 1994 126 6 1527 1536 10.1083/jcb.126.6.1527 8089183 PMC2290961

[B46] Cappelletti G Maggioni MG Tedeschi G Maci R Protein Tyr nitration is triggered by nerve growth factor during neuronal differentiation of PC12 cells Exp Cell Res 2003 288 9 20 10.1016/S0014-4827(03)00209-X 12878155

[B47] Barabasi AL Stanley HE Fractal concepts in surface growth 1995 New York: Cambridge University Press

[B48] Caruso T Lenardi C Agostino RG Amati M Bongiorno G Mazza T Policicchio A Formoso V Maccallini E Colavita E Chiarello G Finetti P Šutara F Skála T Piseri P Prince KC Milani P Electronic structure of cluster assembled nanostructured TiO2 by resonant photoemission at the Ti L2,3 edge J Chem Phys 2008 128 094704 10.1063/1.2832321 18331107

[B49] Caruso T Lenardi C Mazza T Policicchio A Bongiorno G Agostino RG Chiarello G Colavita E Finetti P Prince KC Ducati C Piseri P Milani P Photoemission investigations on nanostructured TiO2 grown by cluster assembling Surf Sci 2007 601 2688 2691 10.1016/j.susc.2006.12.025

[B50] Podestà A Bongiorno G Scopelliti PE Bovio S Milani P Semprebon C Mistura G Cluster-assembled nanostructured titanium oxide films with tailored wettability J Phys Chem 2009 113 18264 18269

[B51] Contestabile A Ciani E Role of nitric oxide in the regulation of neuronal proliferation, survival and differentiation Neurochem Int 2004 45 903 914 10.1016/j.neuint.2004.03.021 15312985

[B52] Hindley S Juurlink BH Gysbers JW Middlemiss PJ Herman MA Rathbone MP Nitric oxide donors enhance neutrophin-induced neurite outgrowth through a cGMP-dependent mechanism J Neurosci Res 1997 45 427 439 9057136

[B53] Yamazaki M Chiba K Mohri T Hatanaka H Cyclic GMP-dependent neurite outgrowth by genipin and nerve growth factor in PC12h cells Eur J Pharmacol 2004 488 35 43 10.1016/j.ejphar.2004.02.009 15044033

[B54] Cappelletti G Tedeschi G Maggioni MG Negri A Nonnis S Maci R The nitration of τ protein in neurone-like PC12 cells FEBS Lett 2004 562 35 39 10.1016/S0014-5793(04)00173-5 15043998

[B55] Nonnis S Cappelletti G Taverna F Ronchi C Ronchi S Negri A Tau is endogenously nitrated in mouse brain: identification of a tyrosine residue modified in vivo by NO Neurochem Res 2008 33 518 525 10.1007/s11064-007-9467-x 17768677

[B56] Tedeschi G Cappelletti G Nonnis S Taverna F Negri A Ronchi C Tyrosine nitration is a novel post-translational modification occurring on the neuronal intermediate filament protein peripherin Neurochem Res 2007 32 433 441 10.1007/s11064-006-9244-2 17268851

[B57] Oess S Icking A Fulton D Govers R Muller-Esterl W Subcellular targeting and trafficking of nitric oxide synthases Biochem J 2006 396 401 409 10.1042/BJ20060321 16722822 PMC1482820

[B58] Fostermann U Boissel JP Kleimert H Expressional control of the“constitutive” isoforms of nitric oxide synthase (NOS I and NOS III) FASEB J 1998 12 773 790 9657518

[B59] Su Y Kondrikov D Block ER Cytoskeletal regulation of nitric oxide synthase Cell Biochem Biophys 2005 43 439 449 10.1385/CBB:43:3:439 16244368

[B60] Kondrikov D Fonseca FV Elms S Fulton D Black SM Block ER β-Actin association with endothelial nitric-oxide synthase modulates nitric oxide and superoxide generation from the enzyme J Biol Chem 2009 285 4319 4327 19946124 10.1074/jbc.M109.063172 PMC2836036

[B61] Liu Z Cao J Ma Q Gao X Ren J Xue Y GPS-YNO2: Computational prediction of tyrosine nitration sites in proteins Mol Biosyst 2011 7 4 1197 1204 10.1039/c0mb00279h 21258675

[B62] Tedeschi G Cappelletti G Negri A Pagliato L Maggioni MG Maci R Characterization of nitroproteome in neuron-like PC12 cells differentiated with nerve growth factor: identification of two nitration sites in α-tubulin Proteomics 2005 5 2422 2432 10.1002/pmic.200401208 15887183

[B63] Cappelletti G Maggioni MG Ronchi C Maci R Tedeschi G Protein tyrosine nitration is associated with cold- and drug- resistant microtubules in neuronal-like PC12 cells Neurosci Lett 2006 401 159 164 10.1016/j.neulet.2006.03.009 16567039

[B64] Naoi M Maruyama W Shamoto-Nagai M Akao Y Tanaka M Oxidative stress in mithocondria: decision to survival and death of neurons in neurodegenerative disorders Mol Neurobiol 2005 31 81 93 10.1385/MN:31:1-3:081 15953813

[B65] Osen-Sand A Catsicas M Staple JK Jones KA Ayala G Knowles J Grenningloh G Catsicas S Inhibition of axonal growth by SNAP-25 antisense oligonucleotides in vitro and in vivo Nature 1993 364 6436 445 448 10.1038/364445a0 8332215

[B66] Vaudry A Stork PJS Lazarovici P Eiden L Signaling pathways for PC12 cell differentiation: making the right connections Science 2002 296 1648 1649 10.1126/science.1071552 12040181

[B67] Zhang Z Tarone G Turner DC Expression of integrin alpha 1 beta 1 is regulated by nerve growth factor and dexamethasone in PC12 cells. Functional consequences for adhesion and neurite outgrowth J Biol Chem 1993 268 5557 5565 8449918

[B68] Danker K Mecahi N Lucka L Reutter W Horstkorte R The small GTPase ras is involved in growth factor-regulated expression of alpha1 integrin subunit in PC12 cells Biol Chem 2001 382 969 972 11501763 10.1515/BC.2001.121

[B69] Ivankovic-Dikic I Gronroos E Blaukat A Barth BU Dikic I Pyk2 and FAK regulate neurite outgrowth induced by growth factors and integrins Nat Cell Biol 2000 2 574 581 10.1038/35023515 10980697

[B70] Cucina A Sterpetti AV Pupelis G Fragale A Lepidi S Cavallaro A Shear stress induces changes in the morphology and cytoskeleton organization of arterial endothelial cells Eur J Vasc Endovasc Surg 1995 9 86 92 10.1016/S1078-5884(05)80230-8 7664019

[B71] Witteck A Yao Y Fechir M Forstermann U Kleinert H Rho protein-mediated changes in the structure of the actin cytoskeleton regulate human inducible NO synthase gene expression Exp Cell Res 2003 287 106 115 10.1016/S0014-4827(03)00129-0 12799187

[B72] Zeng C Morrison AR Disruption of the actin cytoskeleton regulates cytokine-induced iNOS expression Am J Physiol Cell Physiol 2001 281 C932 C940 11502570 10.1152/ajpcell.2001.281.3.C932

[B73] Hattori Y Kasai K Disruption of the actin cytoskeleton up-regulates iNOS expression in vascular smooth muscle cells J Cardiovasc Pharmacol 2004 43 209 213 10.1097/00005344-200402000-00007 14716207

[B74] Marczin N Jilling T Papapetropoulos A Go C Catravas JD Cytoskeleton-dependent activation of the inducible nitric oxide synthase in cultured aortic smooth muscle cells Br J Pharmacol 1996 118 1085 1094 10.1111/j.1476-5381.1996.tb15510.x 8818330 PMC1909607

[B75] Ory S Destaing O Jurdic P Microtubule dynamics differentially regulates Rho and Rac activity and triggers Rho-independent stress fiber formation in macrophage polykaryons Eur J Cell Biol 2002 81 351 362 10.1078/0171-9335-00255 12113476

[B76] Jung HI Shin I Park YM Kang KW Ha KS Colchicine activates actin polymerization by microtubule depolymerization Mol Cells 1997 7 431 437 9264034

[B77] Kajstura J Sowa G Wronska D Induction of DNA synthesis by microtubule depolymerization is mediated by actin filaments Cytobios 1993 76 67 74 8293681

[B78] Zhang JS Kraus WE Turskey GA Stretch-induced nitric oxide modulates mechanical properties of skeletal muscle cells Am J Physiol Cell Physiol 2004 287 C292 C299 10.1152/ajpcell.00018.2004 15044149

[B79] Marechal G Gailly P Effects of nitric oxide on the contraction of skeletal muscle Cell Mol Life Sci 1999 55 1088 1102 10.1007/s000180050359 10442090 PMC11147106

[B80] Gupta SK Vlahakis E Integrin α9β1 mediates enhanced cell migration through nitric oxide synthase activity regulated by Src tyrosine kinase J Cell Sci 2009 122 2043 2054 10.1242/jcs.041632 19470583 PMC2723157

[B81] Maa MC Chang MY Li J Li YY Hsieh MY Yang CJ The iNOS/Src/FAK axis is critical in Toll-like receptor-mediated cell motility in macrophages Biochim Biophys Acta 2011 1813 136 147 10.1016/j.bbamcr.2010.09.004 20849883

[B82] Miyamoto Y Sakai R Maeda C Takata T Ihara H Tsuchiya Y Watanabe Y Nitric oxide promotes nicotine-triggered ERK signaling via redox reactions in PC12 cells Nitric Oxide 2011 23 344 349 21742048 10.1016/j.niox.2011.06.006

[B83] Yamazaki M Chiba K Mohri T Hatanaka H Activation of the mitogen-activated protein kinase cascade through nitric oxide synthesis as a mechanism of neuritogenenic effect of genipin in PC12h cells J Neurochem 2001 79 43 54 10.1046/j.1471-4159.2001.00533.x 11595756

[B84] Mancuso C Capone C Chairomonte Ranieri S Fusco S Calabrese V Bilirubin as an endogenous modulator of neurotrophin redox signaling J Neurosc Res 2008 86 2235 2249 10.1002/jnr.21665 18338802

[B85] Schonhoff CM Bulseco DA Brancho DM Parada LF Ross AH The RAs-ERK pathway is required for the induction of neuronal nitric oxide synthase in differentiating PC12 cells J Neurochem 2001 78 631 639 10.1046/j.1471-4159.2001.00432.x 11483666

[B86] Kalisch BE Demeris CS Ishak M Rylett RJ Modulation of nerve growth factor-induced activation of MAP kinase in PC12 cells by inhibitors of nitric oxide synthase J Neurochem 2003 87 1321 1332 10.1111/j.1471-4159.2003.02057.x 14713289

[B87] Nakata S Tsutsui M Shimokawa H Tamura M Tasaki H Vascular neuronal NO synthase is selectively upregulated by platelet-derived growth factor involvement of the MEK/ERK pathway Arterioscler Thromb Vasc Biol 2005 25 2502 2508 10.1161/01.ATV.0000190663.88143.97 16224055

[B88] Chan SHH Sun EYH Chang AYW Extracellular signal-regulated kinase 1/2 plays a pro-life role in experimental brain stem death via MAPK signal-interacting kinase at rostral ventriolateral medulla J Biomed Sci 2010 17 17 10.1186/1423-0127-17-17 20226096 PMC2848001

[B89] Chrestensen CA McMurry JL Salerno JC MAP kinases bind endothelial nitric oxide synthase FEBS Open Bio 2012 2 51 55 23650581 10.1016/j.fob.2012.02.002 PMC3642102

[B90] Kumar VB Viji RI Kiran MS Sudhakaran PR Negative modulation of eNOS by laminin involving post-translational phosphorylation J Cell Physiol 2009 219 123 131 10.1002/jcp.21659 19097067

[B91] Kan WH Hsu JT Ba ZS Schwacha MG Chen J P38 MAPK-dependent eNOS upregulation is critical for 17beta-estradiol-mediated cardioprotection following trauma-hemorrhage Am J Physiol Heart Circ Physiol 2008 294 H2627 H2636 10.1152/ajpheart.91444.2007 18408136

[B92] Cragg CL MacKinnon JC Kalisch B Nitric oxide sinthase inhibitors modulate nerve-growth-factor mediated activation of Akt ISNR Cell Biol 2012 2012 847974 11 doi:10.5402/2012/847974

